# Does Diabetes Appear in Distinct Phenotypes in Young People? Results of the Diabetes Mellitus Incidence Cohort Registry (DiMelli)

**DOI:** 10.1371/journal.pone.0074339

**Published:** 2013-09-04

**Authors:** Katharina Warncke, Miriam Krasmann, Ramona Puff, Desirée Dunstheimer, Anette-Gabriele Ziegler, Andreas Beyerlein

**Affiliations:** 1 The Institute of Diabetes Research, Helmholtz Zentrum München, and Forschergruppe Diabetes, Klinikum Rechts der Isar, Technische Universität München, Neuherberg, Germany; 2 Forschergruppe Diabetes e.V., Neuherberg, Germany; 3 The Department of Pediatrics, Klinikum Rechts der Isar, Technische Universität München, München, Germany; 4 The Department of Pediatrics 1, Klinikum Augsburg, Augsburg, Germany; St. Vincent's Institute, Australia

## Abstract

**Introduction:**

The diabetes mellitus Incidence Cohort Registry (DiMelli) aims to characterize diabetes phenotypes by immunologic, metabolic, and genetic markers. We classified patients into three groups according to islet autoantibody status and examined whether patients with multiple diabetes-associated autoantibodies, one autoantibody, or without autoantibodies differed with respect to clinical, metabolic, and genetic parameters, including an insulin sensitivity (IS) score based on waist, HbA1c, and triglycerides. We also assessed whether metabolic markers predicted the immune status.

**Materials and Methods:**

As of June 2012, 630 patients in Bavaria, Germany, aged <20 years diagnosed with any type of diabetes within the preceding 6 months were registered in DiMelli. We compared the clinical and laboratory parameters between islet autoantibody status defined patient groups. Parameters showing the strongest associations were included in principal component analysis. Receiver operating characteristic curves were used to assess the ability of the IS Score to predict islet autoantibody status.

**Results:**

Patients with multiple islet autoantibodies, one autoantibody, or without autoantibodies were significantly different in terms of BMI percentile, weight loss before diagnosis, fasting C-peptide (all, *P*<0.001), and IS Score (*P*=0.034). However, principal component analysis revealed no distinct patterns according to autoantibody status. At the optimal IS Score cut-off for predicting islet autoantibody positivity (single compared to none), the specificity was 52.0% and the sensitivity was 86.8%. With respect to prediction of multiple autoantibodies (compared to none), specificity and sensitivity were slightly lower and in combination inferior to those obtained using the BMI percentile and fasting C-peptide.

**Discussion:**

The DiMelli study indicated that patients with and without islet autoantibodies differed with respect to metabolic and genetic markers but there was considerable overlap of phenotypes, and autoantibody status could not be predicted by these parameters. Thus, our results suggest that refined diabetes classification may require both immune and metabolic phenotyping.

## Introduction

The incidence of diabetes in children and adolescents is increasing worldwide [1], a trend which is not expected to stop in the next few decades [2]. Although most patients diagnosed before 20 years of age have type 1 diabetes, there is a considerable number of young patients with type 2 diabetes [3,4], the prevalence of which is increasing in parallel with the number of children with severe obesity [5].

From a clinical perspective, it can be difficult to classify patients as having type 1 or type 2 diabetes. It has also been discussed whether some patients display a mixture of phenotypes of type 1 and type 2 diabetes, which is sometimes referred to as “type 1.5 diabetes” or “double diabetes” [6], and require a therapy addressing insulin loss as well as insulin resistance [7]. In an earlier analysis of the population-based Diabetes Mellitus Incidence Cohort Registry (DiMelli), we suggested that patients could be distinguished based on the presence of multiple islet autoantibodies, one autoantibody, or no autoantibodies [8]. However, it remained unclear whether this classification is clinically useful.

Therefore, we aimed to determine whether the incident cases in these three categories differed in terms of clinical, metabolic and genetic parameters. In addition to established markers, such as C-peptide and BMI, we also examined the use of an Insulin Sensitivity (IS) Score which was recently established using data from the SEARCH study [9]. In particular, we asked whether distinct patterns of these variables would associate with islet autoantibody status defined patient groups.

## Materials and Methods

### Ethics statement

Each patient and/or parent provides written informed consent to participate in the registry. The registry and analyses were approved by the medical ethics committee of Bavaria, Germany (Bayerische Landesaerztekammer, #08043).

### Overview of DiMelli

DiMelli started in 2009 and aims to establish a population-based cohort registry on diabetes incidence in Bavaria, Germany, to characterize diabetes phenotypes based on immunologic, metabolic, and genetic markers. Unlike other diabetes registries in Germany [10–12], in addition to recording clinical/demographic data, DiMelli is collecting patient biomaterials to perform standardized laboratory measurements. The detailed protocol has been reported elsewhere [8].

The registry includes children and young adults residing in Bavaria, Germany, who are diagnosed with diabetes of any type according to American Diabetes Association/World Health Organization criteria [13] at <20 years of age, and who are registered within 6 months of diagnosis. Incident cases are reported state-wide by all pediatric hospitals and primary care practitioners specialized in diabetology. In this study, we analysed data on all patients registered until June 2012.

At the registration of each patient, a blood sample is collected and a structured questionnaire is completed by the local physician at the hospital or the primary care center. The questionnaire includes the date of diagnosis of diabetes, duration of symptoms, weight loss before diagnosis, current medications, history of ketonuria, known autoimmune diseases, family history of diabetes, and demographic factors (e.g., nationality, and education levels of the patients and their parents). Weight, height, waist circumference, hip circumference, and blood pressure are assessed by trained staff (nurses or physicians) in accordance with the instructions given on the questionnaire. The blood sample is sent to the central laboratory of the Institute of Diabetes Research, Helmholtz Center, Munich, by overnight express courier.

### Laboratory measurements

Fasting plasma glucose and ketonuria are measured at each clinic. C-peptide, HbA1c, and triglycerides are measured at the central laboratory (Institute of Diabetes Research, Helmholtz Center, Munich). C-peptide concentrations are measured in aprotinin-stabilized EDTA plasma samples using an automated immunoassay analyzer (AIA 360; Tosoh, San Francisco, CA). HbA1c levels are measured in EDTA samples using a glycohemoglobin analyzer (TOSOH-723 G7; Tosoh). Triglycerides are analyzed using an enzymatic colorimetric test on a cobas 8000^®^ analyzer with a c502 module (Roche Diagnostics, Basel, Switzerland). Human leukocyte antigen (HLA) genotyping is performed by high-resolution sequencing-based typing of exons 2 and 3 of *HLA-DRB1* and *HLA-DQB1*, including heterozygous ambiguity resolution (Conexio Genomics, Fremantle, Western Australia). Blood samples are used to determine autoantibodies to insulin, glutamic acid decarboxylase, insulinoma-associated protein 2, and zinc transporter 8, as previously described [14,15], with the upper limit of normal for each assay corresponding to the 99^th^ percentile of control subjects [16–18].

### Data management and statistical analysis

Incident cases were categorized as patients with two or more islet autoantibodies (multiple islet autoantibodies), one islet autoantibody or no islet autoantibody. Insulin autoantibodies were not included in the analysis if the sample was obtained >14 days after diagnosis, because antibodies to insulin can be induced by exogenous insulin therapy [19].

We calculated sex- and age-specific BMI percentiles based on national reference values [20] and the IS Score as exp (4.64725 - 0.02032 × waist [cm] -0.09779 × HbA1c [%] -0.00235 × triglycerides [mg/dl]) [9], and defined high-risk HLA genotypes using the definition applied for the general population in the TEDDY study [21].

We compared the patients with multiple, one, and no islet autoantibodies in terms of age at onset, fasting glucose, fasting C-peptide, BMI percentile, HbA1c at onset, duration of symptoms, weight loss before diagnosis, triglycerides, IS Score, as well as the rates of severe ketonuria, other autoimmune diseases before diagnosis (celiac disease, autoimmune thyroiditis, vitiligo, or Addison’s disease), high-risk HLA genotypes, family history of type 1 diabetes or any diabetes and insulin dependency. We used the Kruskal–Wallis test to compare continuous variables and the χ^2^ test to compare categorical variables among the three groups. To examine age-specific associations, we also compared these groups separately in patients aged 0–9 and 10–20 years.

To explore whether autoantibody status is associated with specific patterns of clinical outcomes, we calculated the empirical distributions of the continuous variables according to autoantibody status using Gaussian kernel density estimates and performed principal component analyses using BMI percentile, weight loss before diagnosis, fasting C-peptide and IS Score as predictor variables because they showed the strongest associations with autoantibody status. In separate sensitivity analyses, we included HLA risk and age in the analysis to calculate the principal component scores stratified by HLA risk.

To assess the usefulness of insulin sensitivity as measured by the IS Score for predicting islet autoantibody status, we plotted receiver operating characteristic (ROC) curves of the IS Score with islet autoantibody positivity as the outcome, and compared these ROC curves with those generated for BMI percentile, weight loss before diagnosis, and fasting C-peptide. The optimal cut-off values for each ROC curve were defined using the Youden index, as specificity + sensitivity -100% [22].

Correlations between continuous variables were assessed using Pearson’s correlation coefficient. *P*-values of <0.05 were considered statistically significant. All statistical analyses were performed using SPSS 20.0 (Chicago, IL, USA) and R 2.14.1 (http://cran.r-project.org).

## Results

### Patient characteristics and frequency of the autoimmune phenotype

A total of 630 incident cases (54.4% male) were registered in DiMelli between April 2009 and June 2012, with a mean of 16 (range 8–25) cases per month (Figure S1). The median age at diabetes onset was 10.3 years (range 0.8–20.0 years), and 297 (47.1%) subjects were <10 years old. Median time after diagnosis was 9 days. In total, 522 cases (82.9%) were positive for two or more islet autoantibodies, 64 (10.2%) had one, and 44 (7.0%) had no autoantibody. The percentage of autoantibody negative cases increased from 4.4% among cases aged 0–9 years to 9.3% among cases aged 10–20 years.

### Comparison of patients according to their islet autoantibody status

Patients with multiple, one, and no islet autoantibody were significantly different with respect to fasting blood sugar levels (highest in cases with multiple islet autoantibodies), age at onset, fasting C-peptide levels, triglycerides and BMI percentiles (each of the latter highest in autoantibody negative cases). Significantly different values between groups were also observed for weight loss before diagnosis, IS-Score and frequencies of severe ketonuria, high-risk HLA genotype and insulin dependency (each lowest in the autoantibody negative group), while HbA1c levels, duration of symptoms, percentage of known autoimmune diseases before diagnosis and family history did not differ significantly between groups (Table 1). This pattern of results was similar in those aged 10–20 years to that in the total cohort. By contrast, in children aged 0–9 years, only fasting glucose, IS Score and insulin dependency were significantly different among the three groups, and IS Score was highest in autoantibody negative cases (data not shown).

**Table 1:** Clinical and laboratory characteristics of patients with multiple islet autoantibodies, one islet autoantibody, or without islet autoantibodies.

	Available *n*	Multiple islet autoantibodies	One islet autoantibody	No islet autoantibody	*P*
		(*n* = 522)	(*n* = 64)	(*n* = 44)	
Age at diabetes onset (years)	630	10.1 (6.5–13.4)	10.5 (7.3–13.4)	12.0 (8.8–14.2)	0.049
Fasting plasma glucose (mg/dL)	586	137 (111–176)	124 (99–161)	116 (100–147)	0.004
Fasting C-peptide (ng/mL)	599	0.4 (0.2–0.7)	0.5 (0.3–0.9)	0.8 (0.3–1.8)	<0.001
BMI percentile	621	26 (6–57)	41 (9–73)	75 (17–95)	<0.001
HbA1c at onset (mg/dL)	584	10.6 (9.0–12.1)	10.3 (8.3–11.9)	10.2 (8.1–11.8)	0.141
Duration of symptoms (days)	574	30 (19–42)	29 (21–47)	26 (13–37)	0.256
Weight loss before diagnosis (kg)	571	2 (1–5)	2 (1–5)	0 (0–2)	<0.001
Triglycerides (mg/dL)	623	80 (61–104)	80 (61–105)	94 (75–133)	0.026
IS Score	330	8.4 (6.3–10.9)	8.9 (7.0–12.5)	5.7 (4.0–9.6)	0.034
Severe ketonuria (%)	591	58.5	51.7	28.2	<0.001
Known autoimmune diseases before the diagnosis of diabetes (%)	611	4.2	0	4.7	0.26
High-risk HLA type (%)	466	33.6	25.6	8.8	0.008
Relative with type 1 diabetes (%)	622	9.5	11.3	7.1	0.777
Relative with any form of diabetes (%)	622	10.0	11.3	14.3	0.670
Insulin dependency (%)	629	97.3	98.4	72.7	<0.001

Values are expressed as medians with interquartile ranges (25^th^–75^th^ percentile) or as the percentage of cases. Continuous variables were compared by the Kruskal–Wallis test and categorical variables were compared using the χ^2^ test. High-risk HLA genotypes were defined using the definitions for the general population in the TEDDY study [21].

**Figure 1:**
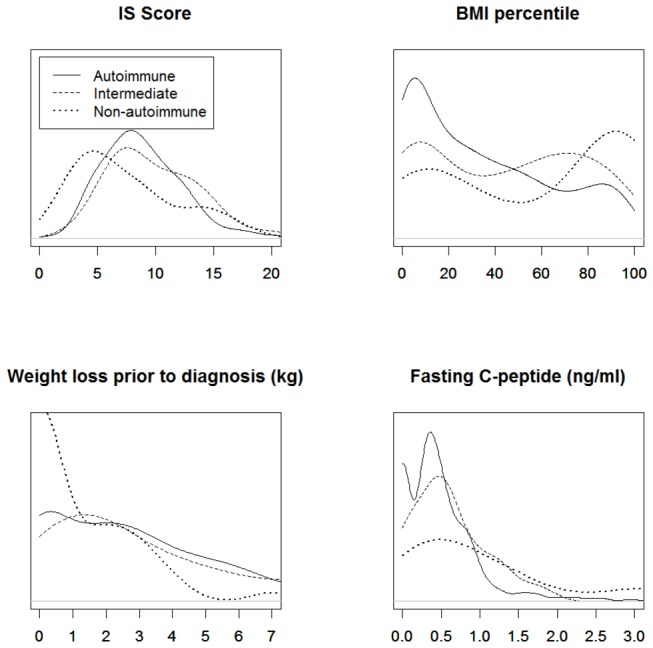
Empirical distributions of Insulin Sensitivity (IS) Score, BMI percentile, weight loss before diagnosis, and fasting C-peptide according to islet autoantibody status.

The strongest associations were observed for BMI percentile, weight loss before diagnosis and fasting C-peptide. There was also a significant association with IS Score, although it was only calculated in about half of the cases. Empirical distribution plots seemed to indicate distinct groups defined by autoantibody status for these four variables (Figure 1), while the plots were less clear for other continuous variables (data not shown).

**Figure 2:**
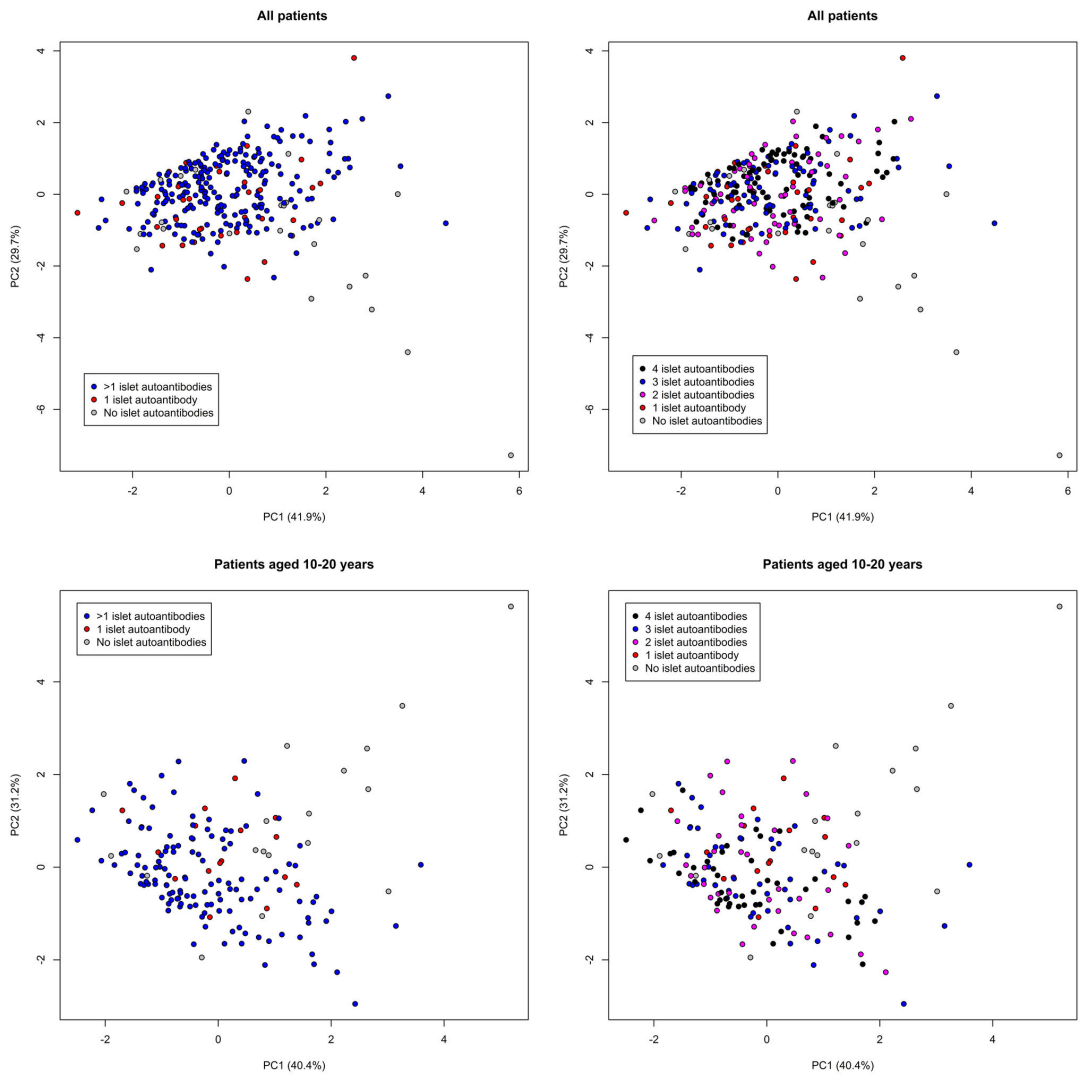
Biplots of the first two principal components (PC) determined by principal component analyses using BMI percentile, weight loss before diagnosis, fasting C-peptide, and insulin sensitivity score, according to the number of autoantibodies in the whole dataset (top) and in children aged 10–20 years (bottom). The variance explained by PC1 and PC2 is shown in parentheses along each axis.

In the principal component analysis based on these four variables, the first two components explained 71.6% of the total variance in the data (Figure 2). The biplot of the first two principal components revealed no clear patterns according to autoantibody status or the number of autoantibodies. Similar results were obtained when the analysis was restricted to patients aged 10–20 years. Six autoantibody negative cases appeared to belong to a relatively isolated pattern in the lower or upper-right regions of the plots, but these accounted for a small proportion of the 44 autoantibody negative cases in the whole cohort (33 were aged 10–20 years). Results were similar when age and HLA risk were included in the analysis, or when we stratified by HLA risk (data not shown).

### Use of the IS Score in DiMelli

**Figure 3:**
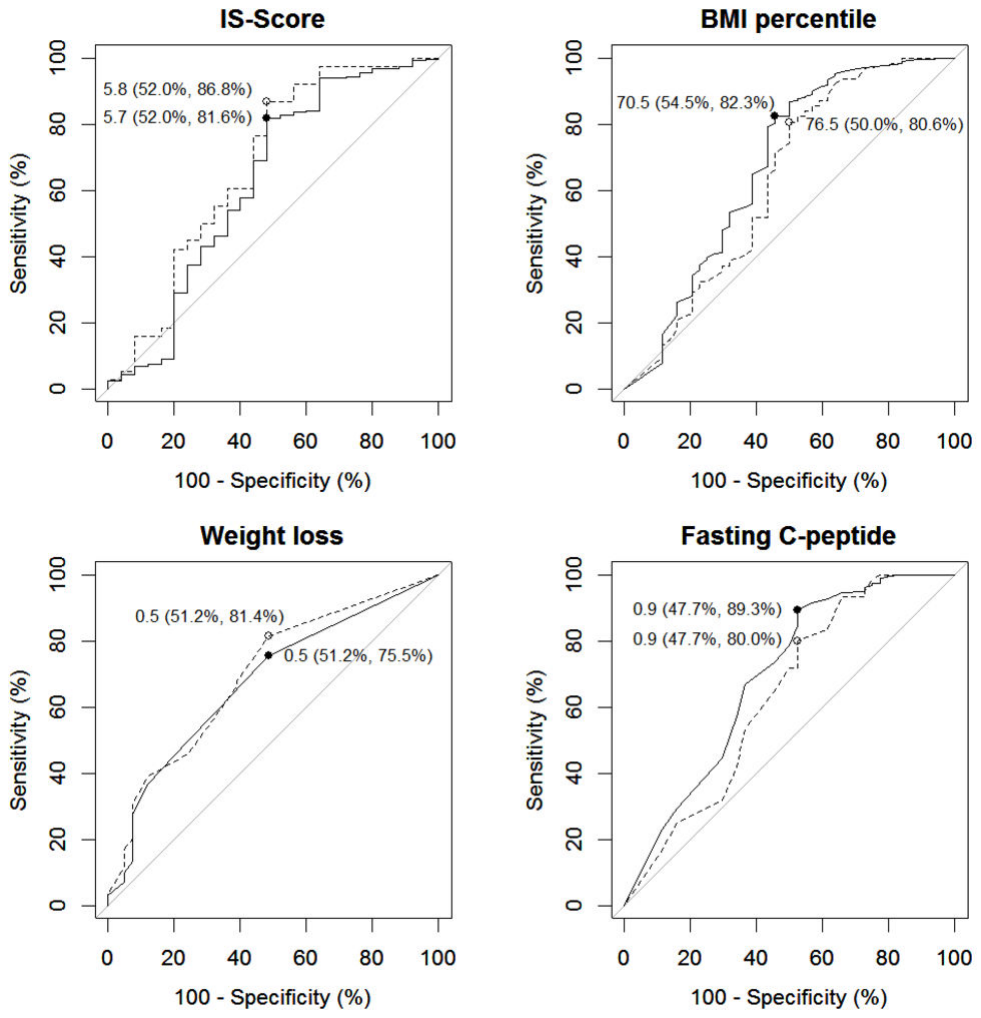
Receiver operating characteristic curves for number of islet autoantibodies status (dashed line: one compared to none, solid line: multiple compared to none) according to Insulin Sensitivity (IS) Score, BMI percentile, weight loss before diagnosis, and fasting C-peptide. Optimal cut-off values determined by the *Youdenindex* with specificity and sensitivity in parentheses are displayed.

The IS Score was inversely correlated with age (*r* = -0.62, *P* < 0.01), BMI percentile (*r* = -0.24, *P* < 0.01) and C-peptide (*r* = -0.15, *P* < 0.01). In ROC analyses, an IS Score of 5.8 was found to be the optimal cut-off for predicting islet autoantibody positivity (one autoantibody compared to none), with a specificity of 52.0% and a sensitivity of 86.8% (Figure 3). When we defined insulin resistance as an IS Score <5.8, we classified 21.2% (n=70) of all patients with available IS Score as insulin resistant. The proportion of patients with insulin resistance was higher in autoantibody negative cases (52.0%, 13/25) than in cases with one islet autoantibody (13.2%, 5/38) and cases with multiple islet autoantibodies (19.5%, 52/267; *P* < 0.001).

The optimal cut-off value for the IS Score offered a better prediction of single islet autoantibody status as defined by a Youden index of 38.8% compared to weight loss before diagnosis (32.6%), BMI percentile (30.6%) and fasting C-peptide (27.7%). However, although the optimal cut-off values for single and multiple autoantibodies were almost identical for all four parameters, BMI percentile (36.9%) and C-peptide (37.0%) had a superior Youden index compared to IS Score (33.6%) with respect to multiple islet autoantibody status.

## Discussion

The present results of DiMelli indicate that this registry can be used to characterize the diabetes phenotypes in children and adolescents. Stable registration numbers confirm that this registry is well accepted by pediatricians and diabetologists, as well as the patients and their parents. The results of our analyses indicate that most incident cases in Bavaria have autoimmune diabetes with multiple islet autoantibodies detected after disease manifestation. The number of cases with one or no autoantibody is also notable, particularly among younger children.

Patients with islet autoantibodies (multiple as well as single) showed clinical characteristics associated with the current description of type 1 diabetes, while the autoantibody negative group more closely resembled what is considered the traditional type 2 diabetes phenotype. In particular, autoantibody negative cases were older and their BMI percentiles, C-peptide levels, and triglyceride levels were higher than those of autoimmune and intermediate cases. These results are consistent with the fact that non-autoimmune diabetes normally occurs later in life, usually after puberty, and is associated with obesity and metabolic syndrome [3,23].

The frequency of high-risk HLA DR-DQ genotypes was considerably lower in children without islet autoantibodies than in children with one or more islet autoantibodies. Nevertheless, only one-third of patients with multiple islet autoantibodies showed high-risk genotypes for type 1 diabetes, suggesting these are no helpful diagnostic criteria for autoimmune diabetes in young children, confirming the results of other studies [24]. Very few patients had other known autoimmune diseases, which is consistent with earlier findings showing that, in most cases of polyendocrinopathy, autoimmune diabetes occurs first, followed by other autoimmune diseases [25].

Although the number of autoantibodies was associated with clinical and laboratory parameters, it remains unanswered whether diabetes has two or more distinct etiologies with separate phenotypes. Principal component analysis did not reveal ‘typical’ combinations of the main distinguishing features that would characterize cases according to their islet autoantibody status. This suggests that the number of autoantibodies is not associated with a distinct pattern of changes in several variables at the same time.

We applied the IS Score developed in the SEARCH study [9] to describe insulin resistance in DiMelli. As a diagnostic tool, the IS Score appears to be rather attractive because it can easily be calculated for any newly diagnosed patient. The parameters used to calculate the IS Score (i.e., HbA1c, triglycerides and waist circumference) are low-cost and are usually routinely analyzed in newly diagnosed patients. We found that most of the cases with a low IS Score (reflecting insulin resistance) were included in the autoantibody negative group, which appears plausible, because autoantibody negative cases are predominantly patients with type 2 diabetes. Unfortunately, the combined specificity and sensitivity of the IS Score at its optimal cut-off value for islet autoimmunity was relatively low and was not superior to those of fasting C-peptide and BMI percentile with respect to prediction of multiple autoantibodies, thus questioning the utility of the IS Score in clinical practice. However, this may apply only to time shortly after diagnosis, when insulin resistance would be expected to be high also in autoantibody positive patients, as their treatment has just started. Indeed, most patients had been recruited in DiMelli within a short time after diagnosis, in contrast to SEARCH, where recruitment was not restricted to a certain time after diagnosis of diabetes and mean time between diagnosis and recruitment was slightly more than one year [26].

Apart from this, DiMelli and SEARCH are similar in their setting, as both are designed as population-based registries with comparable anamnestic, clinical, genetic and laboratory data and outcome definitions and with similar mean recruitment age. In SEARCH, however, autoantibody screening was restricted to two islet autoantibodies (glutamic acid decarboxylase and insulinoma-associated protein 2), while DiMelli includes measurements of four potentially relevant autoantibodies (comprising also autoantibodies to insulin and zinc transporter 8) and thus provides a more detailed assessment of islet autoimmunity.

DiMelli has several strengths. Over the last 3 years, 630 patients were recruited, at a stable rate of approximately 200 patients/year. According to data from the Federal Statistical Office, about 2.43 million people aged <20 years were living in Bavaria at the end of 2010. Based on an estimated incidence of approximately 15 cases/100,000 people/year [10], DiMelli includes >50% of all new cases of diabetes in Bavaria. Thus, DiMelli is an ideal platform for diabetes research in young people. Additionally, unlike other diabetes registries in Europe, all measurements are done in a central laboratory to maintain optimal comparability of laboratory data. Furthermore, four diabetes-associated autoantibodies are measured, including zinc transporter 8 antibody, to optimize the sensitivity of autoantibody testing [27]. Additionally, clinical data are recorded alongside laboratory data.

There are some limitations to the registry. First, DiMelli does not intend to prospectively follow the children with repeated clinical and laboratory examinations, although this may facilitate the classification of diabetes types in some cases. Thus, we cannot preclude that the observed heterogeneity in phenotypes is present only shortly after diagnosis, while more distinguished phenotypes would emerge some years later. Second, the number of autoantibody negative cases is quite low, which limits the statistical power of our analysis.

In conclusion, this analysis of DiMelli revealed that patients with and without islet autoantibodies differed in terms of clinical, metabolic and genetic parameters, but islet autoantibody status did not associate with clear distinct phenotypes based on these other parameters. Furthermore, clinical features and metabolic parameters and their combination were not particularly good at defining autoimmune diabetes. This suggests that refined diabetes classification may require both immune and metabolic testing, with the IS Score being a useful addition to identify insulin resistant phenotypes.

## Supporting Information

Figure S1 **Number of patients registered in DiMelli every month from April 2009 to June 2012.**
(TIF)

Text S1 **Participating clinics and investigators (in alphabetical order).**
(DOCX)
