# Supplementary figures and images for: Does Diabetes Appear in Distinct Phenotypes in Young People? Results of the Diabetes Mellitus Incidence Cohort Registry (DiMelli)
Source: PLoS One. 2013 Sep 4;8(9):e74339. doi: 10.1371/journal.pone.0074339 (PMC3762796; doi:10.1371/journal.pone.0074339)

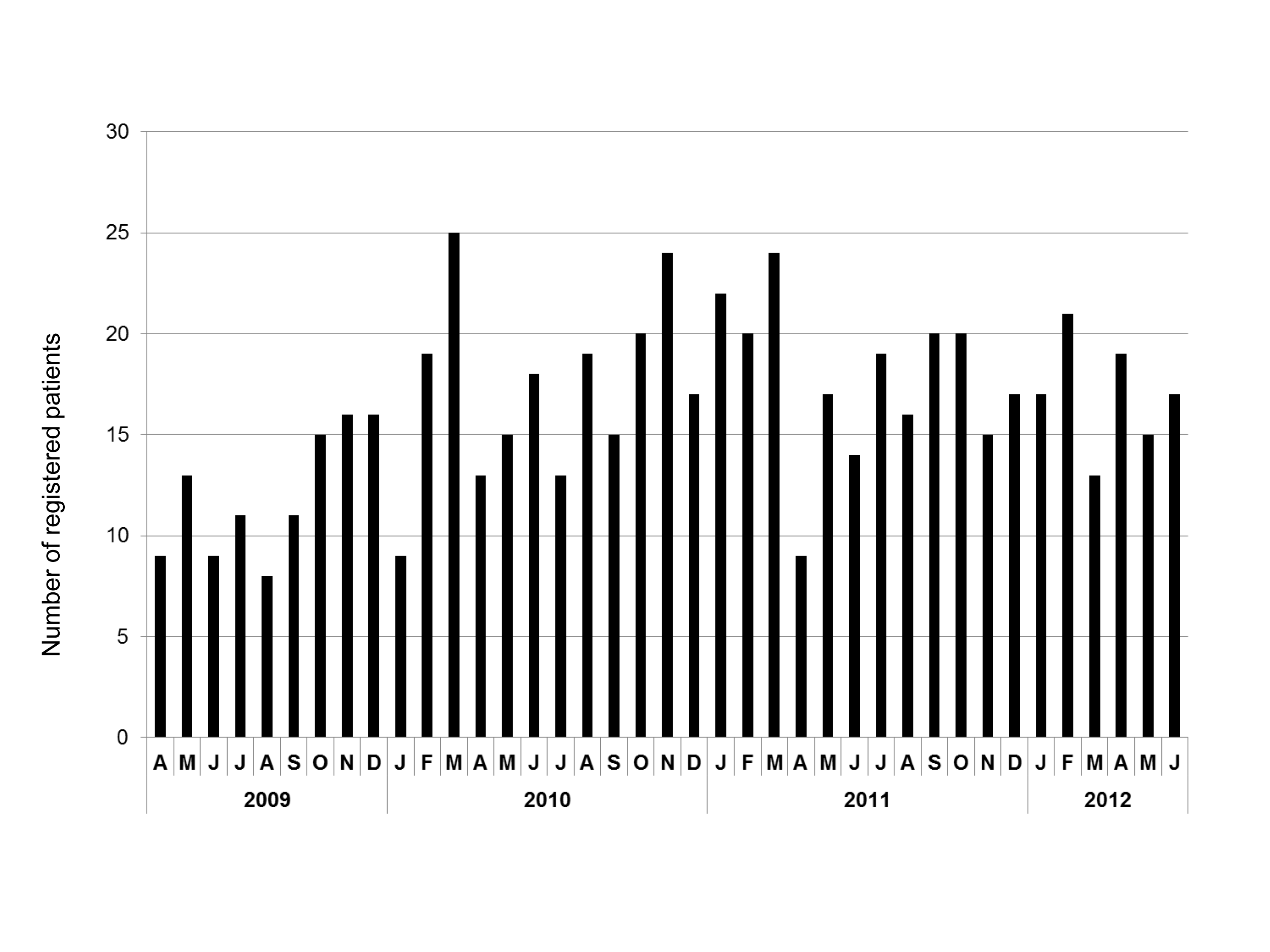

Supplement: Figure S1 — Number of patients registered in DiMelli every month from April 2009 to June 2012. (TIF) [file pone.0074339.s001.tif]
