# Supplementary material for: Does Diabetes Appear in Distinct Phenotypes in Young People? Results of the Diabetes Mellitus Incidence Cohort Registry (DiMelli)
Source: PLoS One. 2013 Sep 4;8(9):e74339. doi: 10.1371/journal.pone.0074339 (PMC3762796; doi:10.1371/journal.pone.0074339)
Supplement: Text S1 — Participating clinics and investigators (in alphabetical order). (DOCX) [file pone.0074339.s002.docx]

**Text S1—Participating clinics and investigators (in alphabetical order):**

Kinderklinik am Klinikum St. Marien Amberg (Katharina Henrich and Theresa Penger), Zentrum für angewandte Diabetologie Asbach-Bäumenheim (Peter Sagemüller), Kinderklinik Aschaffenburg (Eva-Maria Engels), Diabetologische Schwerpunktpraxis Aschaffenburg (Martin Lange and Constantin Sommer), Internistische Gemeinschaftspraxis Aschaffenburg (Martine Klausmann), 1. Klinik für Kinder und Jugendliche am Klinikum Augsburg (Desirée Dunstheimer), Kinderkrankenhaus Josefinum Augsburg (Christian Schlick), Praxis für Kinderheilkunde Bad Kissingen (Juan Carlos Menéndez-Castro), Klinik für Kinder und Jugendliche, Sozialstiftung Bamberg (Meinhard Schatz), Kinderklinik am Klinikum Bayreuth (Marco Wölfel), Praxis für Kinderheilkunde Bayreuth (Katrin Franke-Augustin), Praxis für Kinderheilkunde Bernhardswald (Thomas Eichinger), Klinik für Kinder und Jugendliche am Klinikum Deggendorf (Gerald Beier and Michael Mandl), Internistische Gemeinschaftspraxis Deggendorf (Peter Schräder), Internistische Praxis Eggenfelden (Sabine Merl-Schuh), Praxis für Kinderheilkunde Erlangen (Karsten Naumann), Klinik für Kinder und Jugendliche am Klinikum Fürth (Konrad Freundl and Katja Knab), Sozialpädiatrisches Zentrum Garmisch-Partenkirchen (Petra Vogel-Gerlicher), Ärztezentrum für Kinder- und Jugendmedizin Gauting (Ursula Kuhnle-Krahl), Internistische Praxisgemeinschaft Herrsching (Daniela Petersen-Miecke), Klinik für Kinder und Jugendliche, Sana Klinikum Hof (Susanne Kühner), Internistische Schwerpunktpraxis Diabetologie Holzkirchen (Josef Killer), Internistische Praxis Immenstadt (Walter Glück), Medizinische Klinik 2 am Klinikum Ingolstadt (Peter Zimmer), Internistische Gemeinschaftspraxis Ingolstadt (Peter Hainzinger), Klinik für Kinder- und Jugendmedizin am Klinikum Kempten (Herbert Müller), Gemeinschaftspraxis Bayerwald, Kirchberg (Wolfgang Blank), Kinderarztpraxis Köln (Jochen Schumann), Kinderkrankenhaus St. Marien Landshut (Johannes Hamann), Internistische Praxis Lichtenfels (Ilka Simon-Wagner), Dr. von Haunersches Kinderspital München (Heinrich Schmidt and Susanne Bechtold), Kinderklinik Dritter Orden München (Marina Sindichakis), Kinderklinik München Schwabing, Klinikum Schwabing, StKM GmbH und Klinikum rechts der Isar der Technischen Universität München (Ilse Engelsberger and Katharina Warncke), Kinderarztpraxis München (Birgit Goldschmitt-Wuttge), Allgemeinarztpraxis München (Volker Wallraff), Klinik für Kinder und Jugendliche am Klinikum Nürnberg Süd (Horst Seithe), Kinderklinik Dritter Orden Passau (Eva-Maria Gerstl), Internistische Gemeinschaftspraxis Regensburg (Jochen Arnold), Kinderarztpraxis Regensburg (Peter Gutdeutsch), Klinik für Kinder- und Jugendmedizin am Klinikum Rosenheim (Christian Ockert), Kinderarztpraxis Rothenburg ob der Tauber (Ulrich Zimmer), Kinderklinik am Leopoldina Krankenhaus Schweinfurt (Reinhard Koch), Klinik für Kinder und Jugendliche am Klinikum Starnberg (Robert Tratzmüller), Klinik für Kinder und Jugendliche am Klinikum Traunstein (Doris Schirmer), Klinik für Kinder- und Jugendmedizin der Universität Ulm (Martin Wabitsch and Christian Denzer), Praxis für Allgemeinmedizin Vilshofen (Josef Vöckl), Klinik für Kinder- und Jugendmedizin am Klinikum Weiden (Eva-Maria Haberl), Klinik für Kinder und Jugendliche, Missionsärztliche Klinik Würzburg (Jana Heinze).
